# Genetic structure analysis of cultivated and wild chestnut populations reveals gene flow from cultivars to natural stands

**DOI:** 10.1038/s41598-020-80696-1

**Published:** 2021-01-08

**Authors:** Sogo Nishio, Norio Takada, Shingo Terakami, Yukie Takeuchi, Megumi K. Kimura, Keiya Isoda, Toshihiro Saito, Hiroyuki Iketani

**Affiliations:** 1 Institute of Fruit Tree and Tea Science, NARO, 2-1 Fujimoto, Tsukuba, Ibaraki 305-8605 Japan; 2 Forest Tree Breeding Center, Forestry and Forest Products Research Institute, Juo-cho, Ibaraki, Japan; 3 Faculty of Biosphere-Geosphere Science, Okayama University of Science, Okayama, Okayama Japan

**Keywords:** Plant evolution, Plant domestication, Plant breeding

## Abstract

Japanese chestnut (*Castanea crenata* Sieb. et Zucc.), the only fruit tree species domesticated in Japan, has been cultivated alongside natural stands since prehistorical times. Understanding the genetic diversity of this species and the relationships between cultivated and wild chestnut is important for clarifying its breeding history and determining conservation strategies. We assessed 3 chestnut cultivar populations and 29 wild chestnut populations (618 accessions). Genetic distance analysis revealed that wild populations in the Kyushu region are genetically distant from other populations, whereas other wild and cultivar populations are comparatively similar. Assignment tests suggested that cultivars were relatively similar to populations from central to western Honshu. Bayesian structure analyses showed that wild individuals were roughly classified according to geographical distribution along the Japanese archipelago, except that some wild individuals carried the genetic cluster prevalent in cultivars. Parentage analyses between cultivars and wild individuals identified 26 wild individuals presumed to have a parent–offspring relationship with a cultivar. These results suggested that the genetic structure of some wild individuals in natural stands was influenced by gene flow from cultivars. To conserve wild individuals carrying true “wild” genetic clusters, these individuals should be collected and preserved by ex situ conservation programs.

## Introduction

In plant species, there are large phenotypic differences between cultivated and wild forms. Cultivated plants have been selected by humans for food and other useful materials. Cultivated plants generally have good morphotypes for cultivating and harvesting^1,2^ and bear large fruits or nuts^3–5^. In contrast, wild individuals distributed in various areas have become adapted to different ecological situations, with the result that wild collections derived from divergent areas have greater genetic diversity than do cultivars in some species^6–8^. Thus, wild individuals are interesting for plant breeders as a potential source of genes that have been lost during domestication. The domestication process has been studied in many crops, and several key genes selected during domestication have been identified^3–5,9,10^. Conversely, gene flow from cultivars to wild individuals has influenced the genetic diversity of wild populations of several crops^11–15^. Thus, it is quite important to co-analyze cultivated and wild populations to clarify the genetic history of both population types.

Chestnut utilization in Japan dates back to the Jomon period (14,000–300 BCE). The oldest known nut remains were found in the Omiya-no-moriura ruins (10,900–10,700 BCE), in which carbonized and peeled nuts were discovered^16^. When domestication of Japanese chestnut (*Castanea crenata* Sieb. et Zucc.) began is a difficult but interesting question. Evidence for pre-domestication (called “hansaibai”) of chestnut was found at the location of the Sannai-Maruyama ruins (3500–2000 BCE)^17,18^. The high frequency of chestnut pollen, wood, and nut remains at this site suggested that people preserved chestnut trees around the village. Interestingly, nut size increased during the early to late Jomon period, and the nut size of several remains from the late Jomon period was as large as those of modern cultivars^19,20^, suggesting that the effort to improve genetic performance of Japanese chestnut started prehistorically. The nut size is quite different between cultivars and wild individuals: today, the individual nuts of some local cultivars are more than 30 g, but those of wild individuals are generally less than 5 g (Supplementary Fig. 1). The practice of naming cultivars has existed since the Edo period (1603–1867). The oldest chestnut production area from this period was the Tanba region, which encompasses portions of Kyoto, Osaka, and Hyogo Prefectures in western Japan. Many cultivars with large nuts were thought to have been cultivated in the Tanba region and spread throughout Japan by clonal propagation and seed transportation^21,22^. However, those cultivars and excavated nuts from the Sannai-Maruyama ruins were presumed to be genetically different because there is no genetic information that connects recent cultivars and excavated nuts^23^.

**Figure 1:**
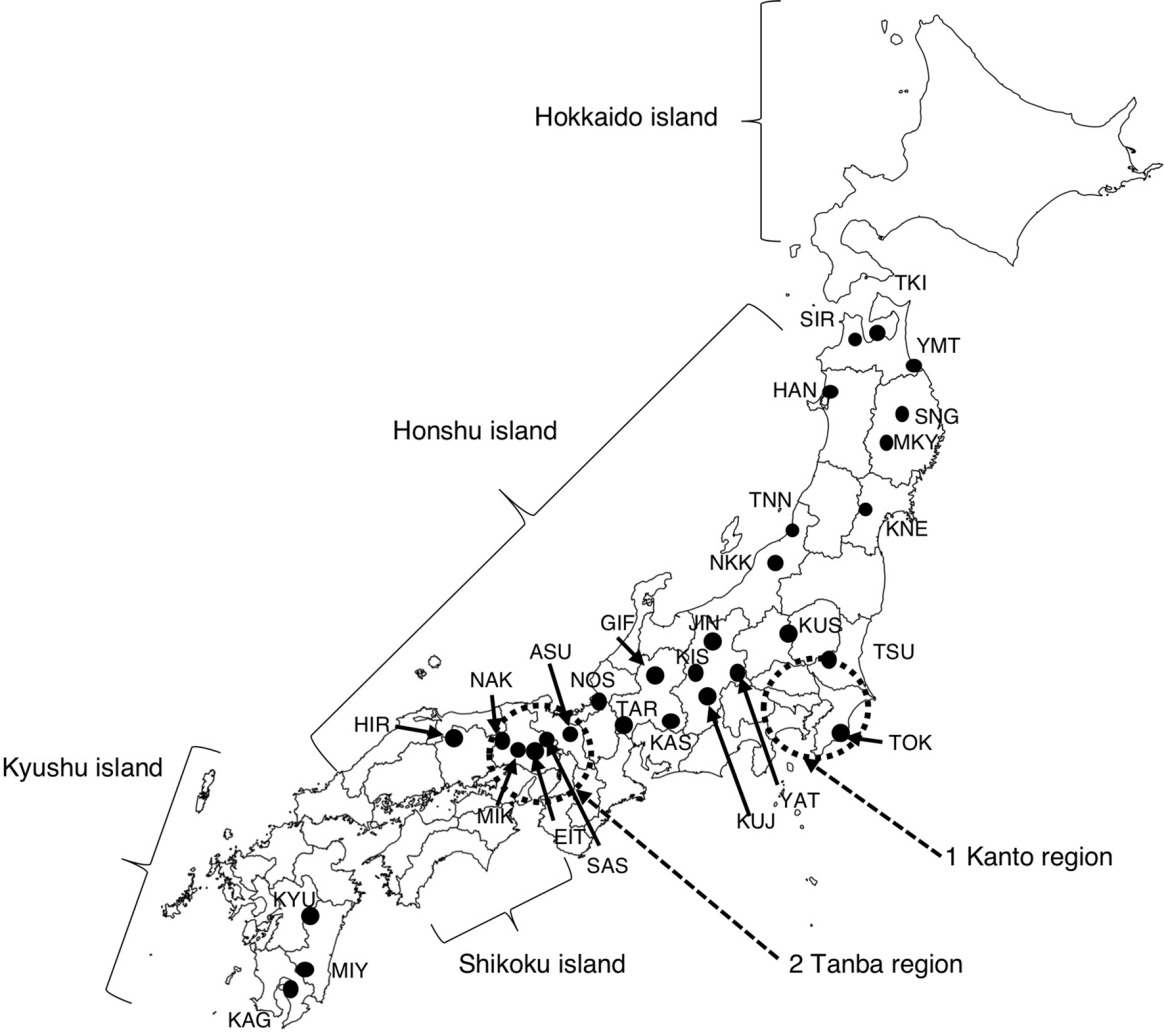
Geographic locations of 3 cultivar populations and 29 wild populations used in this study. The cultivar populations are indicated by dotted circles. Wild populations are indicated by dots. The cultivar population from outside Kanto and Tanba (OR; population number 3) is not shown in this figure because these cultivars are distributed all over Japan. The map was created using MapChart (https://mapchart.net).

The chestnut gall wasp, *Dryocosmus kuriphilus* Yasumatsu, was accidentally introduced from China into Japan around 1940^24^ and has influenced the distribution of both cultivated and wild chestnut. During the 1960s and 1970s, producers cut down local cultivars or naturalized stands and introduced cultivars resistant to chestnut gall wasp. The area of chestnut orchards dramatically increased during that time and was maximized at about 45,000 ha in the late 1970s. On the other hand, wild populations were devastated by chestnut gall wasp until the establishment of *Torymus sinensis* Kamijo, a natural enemy of *Dryocosmus kuriphilus*, during the 1980s. For chestnuts in a natural forest setting, insect pollinators, including bumblebees and flies, disperse pollen across long distances^25^. Even in orchard settings, considerable amounts of pollen are carried in from wild chestnut trees by insect pollinators^26^. Since the 1970s, the number of actively managed chestnut orchards in mountainous areas has gradually decreased due to the inconvenience of country life, so it is possible that seeds from cultivars are carried by animals and may escape from abandoned orchards. Thus, it is important to clarify how much gene flow has occurred from cultivars to wild populations.

Population genetic studies of wild individuals of chestnut have been done using various chestnut species, including European chestnut (*Castanea sativa* Mill.), American chestnut (*Castanea dentata* [Marsh.] Borkh), and Chinese chestnut (*Castanea mollissima* Bl.)^27–32^. In any chestnut species, there are exceptional populations that are genetically different from neighboring populations, suggesting that human-mediated seed dispersal has influenced wild natural stands^28,29,31,32^. So far, however, population genetic studies of Japanese chestnut have been limited to populations from the Tohoku area^33^ of northeast Japan and classifications of cultivars^26,34^, and to our knowledge there are no reports describing genetic characterization of wild chestnut populations throughout Japan.

Chestnut is the only domesticated nut species in family Fagaceae. Several of the deciduous species in family Fagaceae are distributed throughout the Japanese archipelago. The genetic population structures of *Fagus japonica* Maxim., *Quercus aliena* Blume, *Q. serrata* Murray, and *Q. mongolica* var. *crispula* in Japan roughly distinguish northeast and southwest groups^35–37^. For *F. japonica* and *Q. serrata*, the boundary coincides with the north–south trending Itoigawa–Shizuoka Tectonic Line on the western border of Fossa Magna, a great rift valley. On the other hand, data for *Fagus crenata* Blume, a dominant species in cool-temperate deciduous forests in Japan, provide clear evidence of genetic divergence between populations along the Japan Sea (Japan Sea lineage) and Pacific (Pacific lineage) sides of Japan^38^. The nut remains of these species were also discovered from several excavations during the Jomon period, but with the exception of certain *Fagus* species, nuts of these species contain considerable amounts of tannins^39^, which need to be removed before eating. Consequently, the deciduous species in the Fagaceae in Japan other than chestnut have not been domesticated for use as food. Therefore, it is interesting to compare the genetic structures of chestnut and these non-domesticated species.

The objectives of this study were to examine the genetic structure of wild chestnut distributed across the main islands of Japan, to examine the genetic relationships between cultivated and wild populations, and to clarify the breeding history of Japanese chestnut cultivars. Because chestnut orchards are found throughout Japan, gene flow from cultivars to wild individuals should be clarified to support future conservation and breeding strategies.

## Results

### Basic genetic characteristics of chestnut populations

We genotyped chestnuts from 32 populations (618 individuals in total) by using 30 nuclear simple sequence repeat (nSSR) markers (Table 1; Supplementary Tables S1–S3; Fig. 1). Most of the markers had moderate values for genetic diversity (*H*_o_ = 0.687 and *H*_e_ = 0.749 for mean values of 30 nSSR markers). The null allele frequencies ranged from 0.013 (PRD84) to 0.097 (EMCs2; Supplementary Table S3), indicating that the markers are reliable enough to use in population genetic studies. Allelic richness (*A*_*r*_) of the 32 populations ranged from 4.75 to 6.33 (KYU and TAR, respectively; Table 1). The mean *A*_*r*_ value of the cultivar populations (5.46) was similar to that of the wild populations (5.55). Observed and expected heterozygosity of the 32 populations was *H*_o_ = 0.605–0.752 and *H*_e_ = 0.596–0.726, respectively (Table 1). Similar to the *A*_*r*_ values, mean heterozygosities of cultivar populations (*H*_o_ = 0.708 and *H*_e_ = 0.699) were similar to those of wild populations (*H*_o_ = 0.685 and *H*_e_ = 0.681). The inbreeding coefficient (*F*_*is*_) ranged from − 0.034 to 0.077. Cultivar populations originated in regions other than KAN or TAN (designated “other regions”; OR) and six wild populations (SIR, TSU, TOK, YAT, MIK, and NAK) showed significant *F*_*is*_ values (Table 1).

**Table 1:** Grouping and genetic diversity of the 32 chestnut populations investigated in this study.

Population	Group	No. of trees	*N*_*a*_	*A*_*r*_	*H*_*o*_	*H*_*e*_	*F*_*is*_	Chloroplast haplotype	
								HAP1	HAP2
KAN	Cultivar	14	5.6	5.37	0.721	0.689	− 0.011	14	0
TAN	Cultivar	21	6.0	5.28	0.724	0.685	− 0.032	21	0
OR	Cultivar	26	6.9	5.66	0.687	0.716	0.060**	26	0
Mean of cultivar populations			6.3	5.46	0.708	0.699			
TKI	Wild	20	5.5	4.77	0.622	0.620	0.023	20	0
SIR	Wild	20	5.6	5.05	0.625	0.635	0.042*	20	0
YMT	Wild	20	5.5	4.90	0.670	0.649	− 0.007	20	0
HAN	Wild	20	6.2	5.42	0.677	0.668	0.013	20	0
SNG	Wild	20	6.5	5.72	0.693	0.691	0.022	20	0
MKY	Wild	20	6.2	5.53	0.718	0.696	− 0.007	20	0
KNE	Wild	20	6.5	5.56	0.642	0.650	0.038	20	0
TNN	Wild	20	5.8	5.15	0.682	0.688	0.035	20	0
NKK	Wild	20	6.3	5.59	0.718	0.681	− 0.029	20	0
KUS	Wild	20	6.5	5.75	0.710	0.704	0.017	20	0
TSU	Wild	17	5.8	5.33	0.651	0.669	0.057*	17	0
TOK	Wild	21	6.0	5.28	0.673	0.696	0.057**	21	0
YAT	Wild	20	6.3	5.57	0.647	0.673	0.065**	20	0
JIN	Wild	19	6.4	5.78	0.714	0.695	0.000	19	0
KIS	Wild	20	7.0	6.03	0.697	0.698	0.027	20	0
KUJ	Wild	18	6.2	5.60	0.680	0.684	0.035	18	0
GIF	Wild	20	6.1	5.48	0.738	0.697	− 0.034	20	0
KAS	Wild	21	6.8	5.98	0.708	0.711	0.029	21	0
TAR	Wild	17	7.0	6.33	0.741	0.726	0.010	17	0
NOS	Wild	16	6.3	5.93	0.752	0.715	− 0.020	16	0
ASU	Wild	20	6.9	5.95	0.723	0.698	− 0.011	20	0
SAS	Wild	18	6.6	5.97	0.720	0.705	0.006	18	0
EIT	Wild	20	6.6	5.91	0.712	0.701	0.010	20	0
MIK	Wild	20	6.4	5.64	0.663	0.686	0.059**	20	0
NAK	Wild	20	6.6	5.87	0.682	0.719	0.077**	20	0
HIR	Wild	20	6.3	5.54	0.692	0.691	0.025	20	0
KYU	Wild	20	5.4	4.75	0.605	0.596	0.010	0	20
MIY	Wild	18	6.2	5.59	0.691	0.682	0.016	4	14
KAG	Wild	12	5.0	5.03	0.622	0.625	0.047	2	10
Mean of wild populations			6.2	5.55	0.685	0.681			

*N*_a_ number of alleles; *A*_*r*_ allelic richness; *H*_o_ observed heterozygosity; *H*_e_ expected heterozygosity; *F*_is_ fixation index.

*P < 0.05; **P < 0.01.

### Genetic relationship among populations

Chloroplast haplotype frequencies of the 618 individuals were determined using 4 chloroplast SSR markers (cpSSRs). Markers Cmcs1 and Cmcs3 each showed a single fragment size in all individuals, whereas Cmcs2 and Cmcs5 each showed two fragment sizes. Only two chloroplast haplotypes (HAP1 and HAP2; Table 2) were identified among the 618 individuals (Table 1). HAP1 was composed of the combination of 143 bp for Cmcs2 and 151 bp for Cmcs5, whereas HAP2 was composed of 142 bp for Cmcs2 and 149 bp for Cmcs5. The combinations of 143 bp for Cmcs2 and 149 bp for Cmcs5, and of 142 bp for Cmcs2 and 151 bp for Cmcs5, were both completely missing, suggesting that these haplotypes differentiated a long time ago and became geographically separated. All of the cultivars and the 26 wild populations on Honshu carried only HAP1. Within the wild populations in the Kyushu region, population KYU carried only HAP2, whereas MIY and KAG carried both HAP1 and HAP2 (Table 1).

**Table 2:** Chloroplast haplotypes identified in this study.

Chloroplast haplotype	Cmcs1 (KY951992:82981)	Cmcs2 (KY951992:39981)	Cmcs3 (KY951992:69174)	Cmcs5 KY951992:125578)
HAP1	99	143	162	151
HAP2	99	142	162	149

The name of each chloroplast SSR marker is followed by its putative position in the complete *Castanea mollissima* chloroplast genome (GenBank ID:KY951992) in parentheses.

The table values are allele sizes (bp).

Pairwise Jost’s *D* values among the 32 populations were calculated using the 30 nSSRs (Supplementary Table S4). The 3 cultivar populations were genetically close to one another (KAN vs TAN [Jost’s *D* = 0.079], KAN vs OR [0.033], TAN vs OR [0.015]). KAN (a cultivar population) was also close to TOK (a wild population; Jost’s *D* = 0.025), likely because both originated in the same region (see Fig. 1). The 3 wild populations in Kyushu (KYU, MIY, and KAG) were genetically distant from other populations (0.313–0.651; mean 0.460) but relatively close to each other (KYU vs MIY [0.133], KYU vs KAG [0.197], MIY vs KAG [0.123]). Bottleneck tests of heterozygosity excess were also conducted in the 32 chestnut populations. *P* values were significant (< 0.05) for TNN, TOK, NOS, and NAK under the two-phase model (TPM) and for NOS under the stepwise mutation model (SMM) (Supplementary Table S5), indicating that some wild populations have undergone a genetic bottleneck.

### Population structures

**Figure 2:**
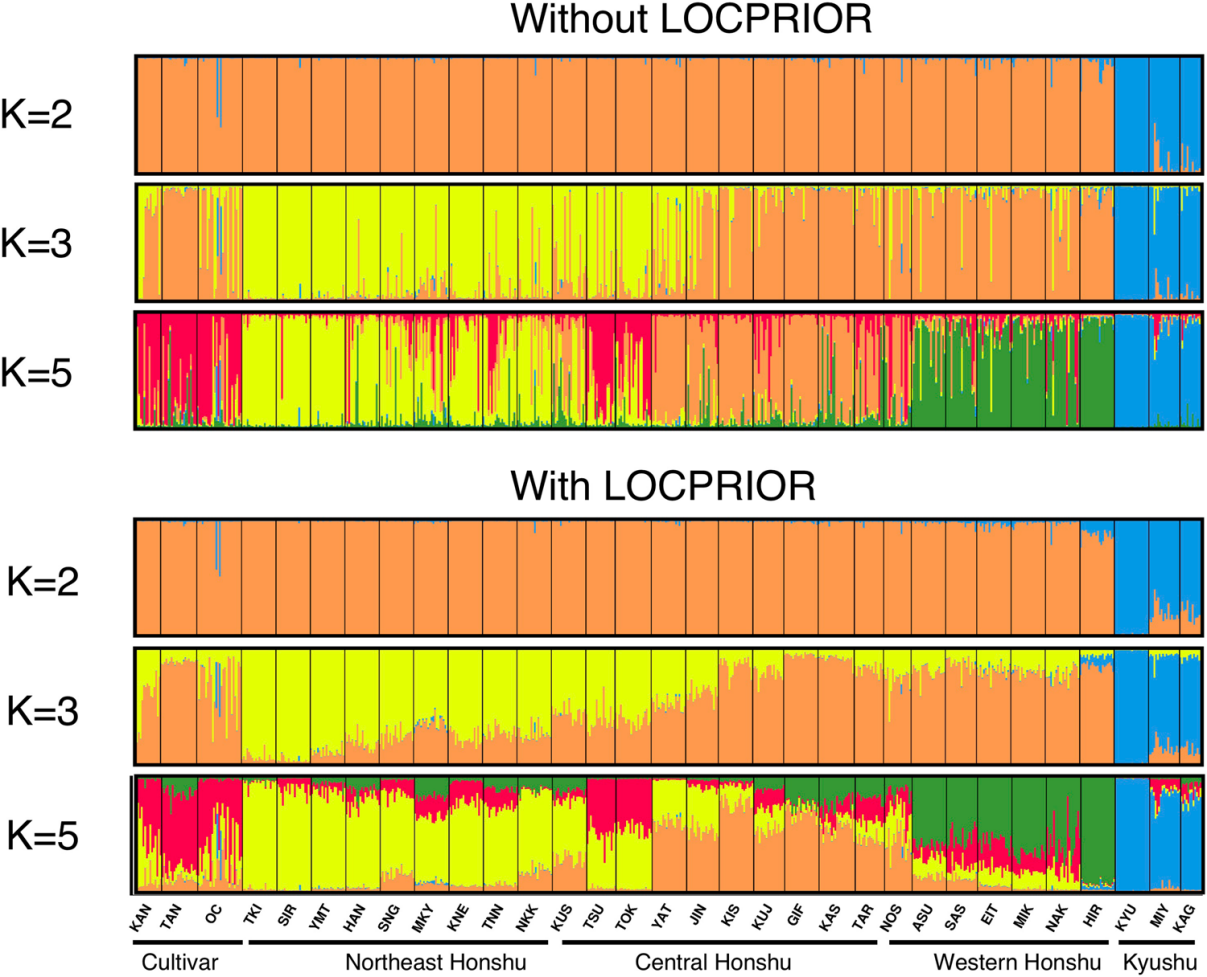
Bayesian estimates of population structure based on nSSR data for the 3 cultivar populations and 29 wild populations. Results for *K* = 2, *K* = 3, and *K* = 5 obtained in STRUCTURE with and without LOCPRIOR are shown for comparison. The CLUMPAK online tool (http://clumpak.tau.ac.il/)^66,67^ was applied to graphically display the results produced by STRUCTURE.

To estimate the optimal number of genetic clusters (*K*) in STRUCTURE, we calculated ∆*K* values (Supplementary Table S6). The ∆*K* values were highest at *K* = 2 both with and without the LOCPRIOR model. In both models, one cluster corresponded to wild populations in the Kyushu region (blue genetic cluster; Fig. 2) and the other cluster to cultivar populations and the wild populations on Honshu island (orange genetic cluster). In both models, the second-highest ∆*K* values occurred at *K* = 3, whereas the values of |L″(K)| and ∆*K* were lower at *K* = 4. The values of |L″(K)| and ∆*K* at *K* = 5 were higher than those at *K* = 4. Because the 3 wild populations in Kyushu were genetically distant from the other 29 populations, we also estimated ∆*K* values using only the 29 populations from outside Kyushu. The ∆*K* values for this subset were clearly higher at *K* = 2 and *K* = 4 (corresponding to *K* = 3 and *K* = 5 using all populations) than at other *K* values, both with and without the LOCPRIOR model. In addition, we applied hierarchal analyses of molecular variance (AMOVA) to estimate the variance among the clusters identified in STRUCTURE (Supplementary Table S7). The results of AMOVA based on two clusters (*K* = 2) across all 32 populations showed that 23.4% of the total variance was among clusters, 6.1% was among populations within clusters, and 70.5% was within populations. Effects both among clusters and among populations within clusters were significant (*P* < 0.001). The percentage of variance among clusters based on five clusters (*K* = 5) in all 32 populations and that based on four clusters (*K* = 4) in the set of 29 populations excluding populations from Kyushu were small (7.8% and 2.8%, respectively) but significant at *P* < 0.001.

**Figure 3:**
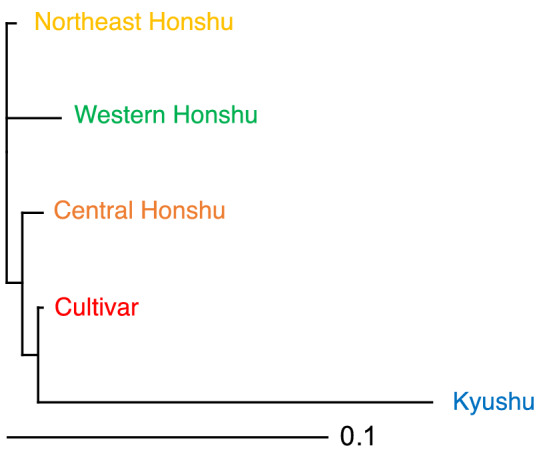
Neighbor-joining tree for the five clusters based on net nucleotide distances for *K* = 5. The colors of the groups were assigned based on the genetic clusters constructed by Bayesian structure analysis for *K* = 5 (see Fig. 2).

Thus, we constructed additional bar plot diagrams for *K* = 3 and *K* = 5 using all 32 populations (Fig. 2) and for *K* = 2 and *K* = 4 using the set of 29 populations excluding populations from Kyushu (Supplementary Fig. S2). The results of the clustering at *K* = 3 and *K* = 5 using all populations and those at *K* = 2 and *K* = 4 using the 29 populations from outside Kyushu were quite similar, so we focused on the clustering results obtained for the full set of populations. The results of the clustering at *K* = 3 with and without LOCPRIOR were similar. In each case, the accessions were classified into three clusters corresponding to (1) the cultivar populations and the wild populations on central and western Honshu island (“orange”), (2) wild populations on northeast Honshu island (“yellow”), and (3) wild populations in the Kyushu region (“blue”). When *K* = 5 with and without LOCPRIOR, we further identified a “red” genetic cluster corresponding to the cultivar populations and a “green” one corresponding to wild populations on western Honshu island. Hereafter, the “red”, “yellow”, “orange”, “green”, and “blue” genetic clusters at *K* = 5 are referred to as the “cultivar”, “northeast Honshu”, “central Honshu”, “western Honshu”, and “Kyushu” clusters, respectively. A neighbor-joining tree was constructed based on the net nucleotide distances between pairs of clusters (Fig. 3). The “cultivar” cluster was genetically similar to the “northeast Honshu”, “central Honshu”, and “western Honshu” clusters, whereas the “Kyushu” cluster was genetically quite distant from the other four clusters. A noticeable contribution of the “cultivar” cluster was observed in some wild individuals in regions ranging from Tohoku to Kyushu. In particular, TSU and TOK (wild populations near Tokyo) carried a high proportion of the “cultivar” cluster (68.3% for TSU and 51.4% for TOK when analyzed without the LOCPRIOR model).

To validate the genetic structures of wild chestnut populations in Japan, 95 wild chestnut individuals collected by the Forest Tree Breeding Center (FTBC) were combined with the 32 populations in a Bayesian structure analysis without the LOCPRIOR model (Supplementary Table S8). The results were quite similar to those based only on the 32 populations for *K* = 2, 3, or 5 in that the wild populations were differentiated by region from northeast to southwest, with the exception that the “cultivar” cluster was highly represented in individuals from Akita and Okayama (which are not geographically close) when *K* = 5 (Supplementary Fig. S3). For *K* = 5, individuals derived from northeast Honshu (Aomori, Akita, and Miyagi) mainly showed the “northeast Honshu” cluster, whereas those from the western end of Honshu (Tottori, Shimane, Yamaguchi, and Hiroshima), which was not well represented in the analysis of 32 populations, mainly showed the “western Honshu” cluster. The FTBC collection did not include individuals originated in Kyushu, but a noticeable contribution of the “Kyushu” cluster was observed in FTBC individuals derived from Tottori, Shimane, and Yamaguchi, which are geographically close to Kyushu.

### Parentage analysis

To clarify whether cultivars had influenced the genetic structure of wild individuals, we conducted parentage analysis of 557 wild individuals (Table 1) by using 92 major cultivars (Supplementary Table S1) as a putative parent database. We identified 26 wild individuals presumed to have a direct offspring–parent relationship with a cultivar in the database (Table 3). The delta score for putative parents ranged from 1.7 to 19.5. Traditional cultivar ‘Ginyose’ was presumed to be a parent of wild individuals TSU11, NAK13, and NAK17. Recent major cultivar ‘Tsukuba’ was also found to be a putative parent of HAN01, NOS14, and NOS15. Another major cultivar, ‘Ishizuchi’, was found to be a putative parent of KNE02 and TOK3. We also conducted parentage analysis of wild individuals collected by FTBC (Supplementary Table S8) using the database of 92 major cultivars and identified 8 putative parent–offspring pairs between cultivars and wild individuals (Supplementary Table S9). The local cultivars ‘Saimyouji 1′ and/or ‘Saimyouji 2′ were presumed to be a parent of 3 wild individuals from Akita. In addition, 3 wild individuals from Okayama were presumed to be offspring of the traditional cultivar ‘Ginyose’ or of ‘Hiratsuka 4′, a selection from ‘Ginyose’.

**Table 3:** Putative parent cultivars of wild individuals identified using CERVUS software.

Location	Population	Genotype	Putative parent cultivar	Delta
Akita	HAN	HAN01	Tsukuba	7.3
Akita	HAN	HAN03	Moriwase	15.1
Iwate	MKY	MKY08	Buzen	2.9
Miyagi	KNE	KNE02	Ishizuchi	3.4
Nigata	TNN	TNN06	Moriwase	15.9
Nigata	NKK	NKK20	Kinshuu	11.5
Ibaraki	TSU	TSU6	Nakatetanba	5.2
Ibaraki	TSU	TSU11	Ginyose	2.5
Chiba	TOK	TOK3	Ishizuchi	10.6
Chiba	TOK	TOK4	Tajiriginyose	7.0
Gifu	TAR	TAR15	Tamatsukuri	7.9
Fukui	NOS	NOS05	Moriwase	14.3
Fukui	NOS	NOS06	Moriwase	19.5
Fukui	NOS	NOS14	Tsukuba	14.9
Fukui	NOS	NOS15	Tsukuba	4.7
Fukui	NOS	NOS16	Obuse 2	3.9
Hyogo	EIT	EIT11	Kinseki	13.3
Hyogo	EIT	EIT20	Imakita	9.2
Hyogo	MIK	MIK7	Arima	15.0
Hyogo	MIK	MIK8	Dengorou	8.5
Hyogo	NAK	NAK13	Ginyose	3.4
Hyogo	NAK	NAK16	Arima	15.1
Hyogo	NAK	NAK17	Ginyose	2.5
Hyogo	NAK	NAK18	Kanotsume	9.5
Miyazaki	MIY	MIY5	Waseginzen	9.7
Miyazaki	MIY	MIY10	Obiwase	1.7

### Assignment of cultivars to wild populations

**Figure 4:**
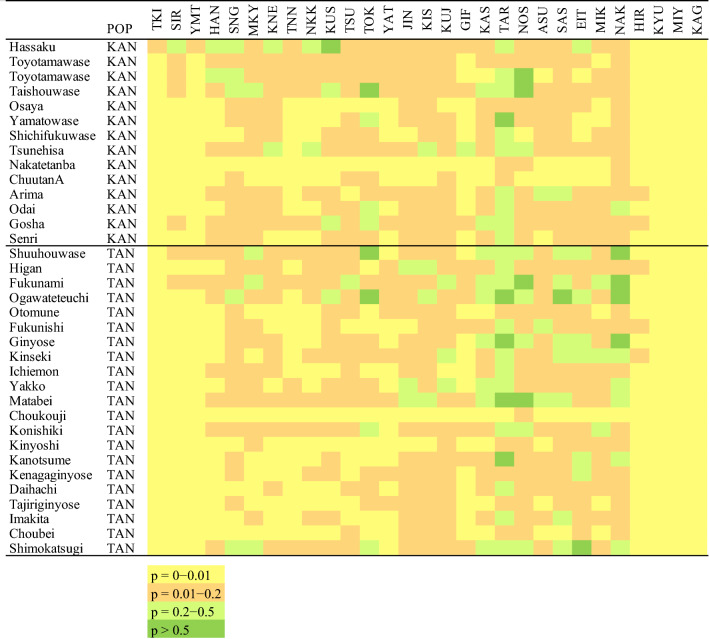
Heatmap showing the probabilities of cultivars from KAN and TAN having originated in each wild population, estimated by an exclusion test.

To infer the origins of the cultivars in this study, 61 cultivars from KAN, TAN, and OR were assigned to the 29 wild populations using GeneClass 2.0. The probabilities of membership are reported in Fig. 4 (KAN and TAN) and Supplementary Table S10 (KAN, TAN, and OR). Overall, cultivars showed higher probabilities of origin in central-to-western populations than in northern and Kyushu populations. In particular, cultivars from the Tanba region were likely to be assigned to central and western populations. For most cultivars, the probabilities of originating from wild populations at the northern end of Honshu and from wild populations in the Kyushu region were zero or nearly zero. The populations TOK, KAS, TAR, NOS, SAS, EIT, and NAK showed higher probability scores than the other populations.

### Analysis of isolation by distance

**Figure 5:**
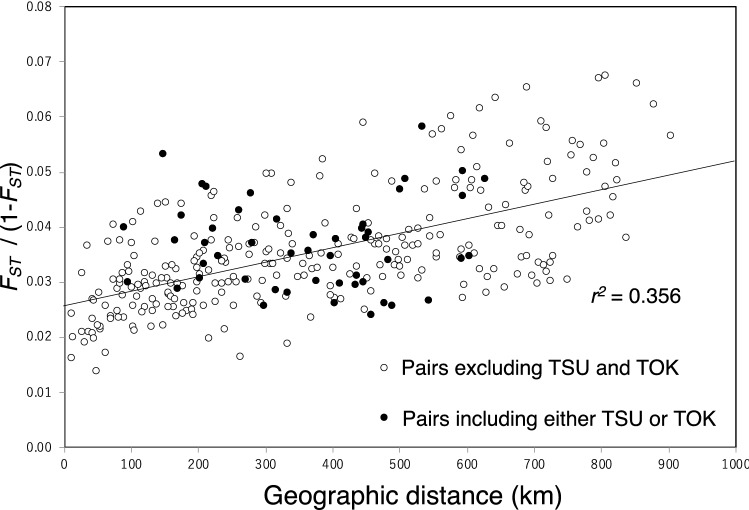
Analysis of isolation by distance among the 26 populations on Honshu island based on a Mantel test with 9999 random permutations. White dots indicate pairs without TSU or TOK, whereas black dots indicate pairs including either TSU or TOK The Mantel test based on all pairs showed significant correlation between genetic and geographic distances (*r*^2^ = 0.356, *p* = 0.001).

To clarify the correlation between genetic differentiation and geographical distance in wild populations, the 26 wild populations on Honshu were used for isolation-by-distance analysis. A significant correlation between genetic distance and geographic distance was detected by the Mantel test when all possible pairs of the 26 wild populations were included (*r*^2^ = 0.356, *P* < 0.001; Fig. 5). When the analysis was limited to pairs that included either TSU or TOK, both of which are closely related to cultivars, there was no significant correlation between genetic differentiation and geographical distance (*r*^2^ = 0.002, *P* < 0.736).

## Discussion

In this study, we examined the genetic diversity and genetic relationships among 29 wild populations and 3 cultivated populations. Genetic diversity parameters such as heterozygosity and allelic richness were similar in cultivated and wild populations, and no significant evidence of genetic bottleneck was identified in cultivated populations. In perennial fruit crops, some species maintained high levels of genetic diversity during domestication, such as apple and grape^40,41^, whereas Chinese cherry showed moderate loss of genetic diversity in cultivated accessions^8^. This kind of comparison depends on the geographic diversity of the cultivars and individuals collected. We identified a total of 242 alleles from 61 cultivars and 385 alleles from 557 wild individuals. Although the number of cultivars actually being propagated in orchards in Japan is less than about 50, the number of wild individuals available is enormous because they are naturally distributed all over Japan. Thus, it should be possible to broaden the genetic diversity of cultivars by introducing favorable alleles from wild populations.

The wild populations in Kyushu are genetically distant from those in other regions and from the cultivar populations (Table 1, Fig. 3). In Bayesian structure analysis using *K* = 3 (Fig. 2), accessions were classified into (1) wild populations in northeast Honshu (“yellow”), (2) cultivars (regardless of location) and wild populations in central and western Honshu (“orange”), and (3) wild populations in the Kyushu region (“blue”). Interestingly, in some individuals from Kyushu populations MIY and KAG, we identified introgressions of genetic clusters from Honshu (“yellow” and “orange”) into the genetic cluster from Kyushu (“blue”), suggesting that seeds and scions have been imported into Kyushu from Honshu island. Human-mediated seed and scion dispersal have been common in chestnut^26,28,29,31,32^. The altitude of MIY is 109–212 m above sea level (asl) and MIY is located close to a Satoyama landscape (secondary woodlands and grasslands adjacent to human settlement)^42^, where human activities are presumed to have had an impact on the distribution of trees. A similar result was obtained from chloroplast haplotype analysis, as MIY and KAG carried not only HAP2 (presumed to have originated in Kyushu) but also HAP1 (Table 1).

The genetic diversity of KYU was lowest among the populations used in this study: this population had only the “Kyushu” genetic cluster and carried only HAP2. The “Kyushu” genetic cluster was rarely observed in cultivars except for ‘Obiwase’ in Miyazaki Prefecture and ‘Nanatate’ in Kochi Prefecture (both from population OR). As the altitude of KYU is high (1037–1074 m asl), it might have had less chance to receive chestnut seed or pollen from other regions and cultivated populations. The “Kyushu” genetic cluster was also detected at low levels in some wild chestnut individuals collected by FTBC in Yamaguchi, Shimane, Hiroshima, and Tottori Prefectures, which are located on the western end of Honshu close to Kyushu island (Supplementary Fig. S2). In a previous study, HAP2 was also identified in Korean cultivar ‘Pochun B-1′^43^. As the Korean peninsula is located not far north of Kyushu island, it may be possible that wild chestnuts in Korea have a genetic structure similar to those on Kyushu island.

With the exception of those in Kyushu, the wild populations were genetically similar and carried only HAP1 (Fig. 3; Table 1). On Honshu island, isolation-by-distance analysis showed continuous patterns of genetic differentiation from one end to the other. Genetic diversity tends to decrease from south to north in the Northern Hemisphere, as is common in deciduous tree species^44^. On the other hand, Bayesian structure analysis with and without LOCPRIOR showed a clear relationship between genetic structure and geographical distribution (Fig. 2). For *K* = 3, wild chestnut populations on Honshu island were divided at the middle of central Honshu, roughly corresponding to the boundary of Fossa Magna, a major tectonic depression. Genetic diversification related to Fossa Magna was also reported in *F. japonica*^35^, *Q. mongolica* var. *crispula*^37^, and *Q. serrata*^36,37^. The genetic boundary of *Q*. *aliena* showed a rather different pattern and was located between Chubu and Kinki Districts, about 200 km west of Fossa Magna^36^. Following yet another pattern, *F. crenata* showed genetic divergence between the Japan Sea lineage and the Pacific lineage^38^. *Quercus variabilis* and *Q. phillyraeoides*, which are mainly distributed south of Fossa Magna, were presumed to be unaffected by this boundary. Also, *Q. acutissima* Carruth is not thought to have been present in the main archipelago of Japan during the last glacial maximum^45^ and shows monomorphism of cpDNA in Japan^46^, suggesting that it was heavily influenced by human activity rather than by natural migration. To summarize these results, Fossa Magna commonly affected the genetic diversification of deciduous species in family Fagaceae that were naturally and widely distributed across this boundary, except for *Q. aliena* and *F. crenata*. The barrier caused by high mountain ranges and large deposits of volcanic rocks might have prevented the migration of seed^37^.

For *K* = 5 in the Bayesian structure analysis, the cultivar populations shared the “red” (“cultivar”) genetic cluster with some wild chestnut populations, especially in the Kanto region (TOK and TSU; Fig. 2). We suspect that the “red” cluster in these wild populations is related to gene flow from cultivars to natural stands. So far, there have been no reports that cultivars were selected from wild individuals in the Kanto region (TOK and TSU), whereas several reports suggest that cultivars were selected in the Tanba region of western Honshu^21,22^. Although genetic differentiation and geological distance among wild populations on Honshu are significantly correlated (*r*^*2*^ = 0.356; Fig. 5), analysis of pairs that included TOK or TSU showed no significant correlation (*r*^*2*^ = 0.002), suggesting that these populations were affected by non-natural (i.e., human-mediated) transportation of genetic clusters from other regions. Furthermore, in Ibaraki Prefecture (the location of TSU), chestnut production and the presence of orchards dramatically increased from the 1940s to the 1960s, as wild individuals were replaced by cultivars^21^. Trees in these orchards might have hybridized with wild individuals in TSU. The direct parent–offspring relationships between cultivars and wild individuals identified in several regions also suggest gene flow from cultivars to wild populations in these regions (Table 3, Supplementary Table S10). In fact, the “cultivar” (“red”) genetic cluster was observed in some wild individuals in regions ranging from Tohoku to Kyushu (TSU, TOK, MKY, KNE, TNN, KUJ, TAR, NOS, SAS, EIT, MIK, NAK, MIY, and others; Fig. 2) when analyzed both with and without the LOCPRIOR model, suggesting that the present wild chestnut populations throughout Japan have been influenced by gene flow from cultivars. Interestingly, all of the wild populations showing significant values in bottleneck analysis under TPM (TNN, TOK, NOS, and NAK) carried considerable portions of the “cultivar” genetic cluster. These sites might have been somewhat influenced by human activities. Large amounts of timber from wild chestnut were used for railway construction^47^ in the early twentieth century. Chestnut timber is thought to have been in short supply by the mid-1910s^48^, and it was reported that trees distributed in forests near railway lines and other convenient places had been cut down and exhausted for railway construction^49^.

Gene flow from cultivars to wild populations has been identified in some fruit tree species. In the Japanese archipelago, pear shows extensive introgression of prehistorically naturalized *P. pyrifolia* into the threatened native species *P. ussuriensis*, which was identified in northern Tohoku^12^. With rare exceptions, wild pear trees have only been found in human-disturbed secondary forests or pasturelands, so native individuals have likely sustained a genetic influence from cultivated or escaped trees. Therefore, true native *P. ussuriensis* is distributed in limited areas and is endangered. In apple, some studies have investigated crop-to-wild gene flow from modern cultivars (*Malus domestica* Borkh.) imported from western countries into native species (*Malus sieversii* and *Malus orientalis)*^15^*.* In grape, a wild population in Georgia had a high proportion of hermaphrodite vines, a character specific to cultivar genotypes, indicating gene flow from cultivated to wild types^50^.

Genetic studies of current chestnut populations in combination with information from preserved nut remnants can reveal clues as to how and where chestnut might have been domesticated. It is obvious that one component of the domestication syndrome in Japanese chestnut is large nut (seed) size, as in other species^51^. Although previous reports clarified that nut size increased during the early to late Jomon (4000–1000 BCE)^17,18^, Motoki suggested that the nuts discovered mainly in ruins in northeast Japan were not directly influenced by native cultivars that originated in the Tanba region, which is geographically far from northeast Japan^23^. In Bayesian structure analysis with *K* = 3, cultivars were closer to wild populations in central to western Honshu, the latter including the Tanba region, than to those in northeast Honshu, which includes Sannai-Maruyama ruin (Aomori Prefecture), Aota ruin, and Yachi ruin (Niigata Prefecture), where large nuts were excavated. Thus, cultivars from the Tanba region would not have been selected directly from the nuts from these ruins. On the other hand, the results of Bayesian analysis and an assignment test suggested that the candidate origin of the cultivars was central to western Honshu, though the Tanba region (western Honshu)is more likely, based on the literature^21,22^. In any case, it is quite difficult to clarify the detailed origin of cultivars by comparisons to wild populations that were influenced by gene flow from cultivars, because the direction of gene flow is opposite to that typically seen during breeding and domestication processes (i.e., from wild populations into cultivars).

In cereals, it took several 1000 years to consistently obtain the non-shattering spikelet phenotype, a defining characteristic of domesticated crops^45^. Among fruit tree species, apple is a good example of a species whose domestication appears to have taken more than a few 1000 years. The cultivated species *M. domestica* originated in the Tian Shan mountains from *M. sieversii*. During the dispersal of apple from Asia to Europe along the Silk Route, hybridization with and introgression from Caucasian and European *Malus* species contributed to form present-day apple cultivars^52^. Slow domestication of a species suggests that there were periods of pre-domestication cultivation^53^, or “hansaibai”, which is suggested by Kitagawa and Yasuda^17^ to have occurred in Japanese chestnut at the location of the Sannai-Maruyama ruin. It is possible that the effort to improve genotypes via pre-domestication cultivation and domestication of chestnut was initiated more than once, and in several regions, because large nut remains were discovered in several ruins from different eras.

In conclusion, this study of both cultivars and wild populations of chestnut indicates the presence of gene flow from cultivars to wild forms and reveals a possible history of cultivar breeding. The genetic relationships between cultivars and wild individuals clarified in this study will be useful for both conservation and breeding. From the perspective of chestnut breeding, wild individuals distributed in Kyushu could be valuable genetic resources because they are genetically quite distant from current cultivars. Climate change is bringing new problems of black spot nut and nut rot to chestnut, and the materials from Kyushu might be helpful for chestnut production in hot areas. For the purposes of conservation, it is important to preserve wild individuals from Kyushu that are divergent from cultivars because gene flow from cultivars to natural stands was observed in this study. To achieve this goal, wild individuals carrying true “wild” genetic clusters should be collected and preserved by ex situ conservation programs such as the gene banks of public institutions.

## Materials and methods

### Plant materials and DNA extraction

Thirty-two populations (618 accessions), including 3 cultivated populations and 29 wild populations, were used in this study (Table 1, Supplementary Tables S1 and S2, and Fig. 1). The three cultivated populations were composed of local cultivars originated in the Kanto region (KAN), Tanba region (TAN), and other regions (OR). All of the sampling sites for wild populations excluded chestnut orchards. Individual trees were selected at least 20 m apart so as to minimize the sampling of close relatives. The heights of most trees were 5 to 15 m, which is typical for wild trees of *Castanea crenata* because damage from the white-striped longicorn beetle (*Batocera lineolata*) shortens the life of *C. crenata* in Japan.

Ninety-five wild chestnut individuals collected by the FTBC as part of a forest tree gene bank program were used to validate the genetic structure of wild chestnut populations (Supplementary Table S7). The FTBC program aimed to collect excellent trees for wood products, so the collections would likely be genetically biased, but they included the western part of Japan (Tottori, Shimane, Hiroshima, and Yamaguchi), where sampling of wild chestnut populations had not previously been done. We did not include this collection in the main analysis because of the potential for bias and the low number of individuals.

Genomic DNA was extracted from young leaves and buds with a DNeasy Plant Mini Kit (Qiagen, Germany) according to the manufacturer’s instructions.

### Genetic markers

The 618 accessions and the wild chestnut individuals collected by FTBC were genotyped for 30 nSSRs^54–56^ (Supplementary Table S3) and 4 cpSSRs^57^ (Cmcs1, Cmcs2, Cmcs3, and Cmcs5; Table 2). PCR amplification was performed in 10 µL containing 5 µL of 2 × Green GoTaq G2 Hot Start Master Mix (0.4 mM each dNTP, Taq DNA polymerase, and 4 mM MgCl_2_, pH 8.5; Promega, Madison, WI, USA), 20 pmol of each forward primer labeled with a fluorescent dye (5-FAM or 5-HEX) and unlabeled reverse primer, and 2.5 ng of genomic DNA. Amplification was performed in 35 cycles of 94 °C for 1 min, 55 °C for 1 min, and 72 °C for 2 min. PCR products were separated and detected with a 3130xl Genetic Analyzer (Life Technologies, Carlsbad, CA, USA). The size of each amplified band was determined by comparison with a set of internal-standard DNA fragments (400HD ROX, Life Technologies) in GeneMapper software v. 5.0 (Life Technologies).

### Basic genetic characteristics

The observed heterozygosity (*H*_o_) and expected heterozygosity (*H*_e_) were calculated in GenAlEx v. 6.5 software^58^, and inbreeding coefficients (*F*_*is*_) and allelic richness (*A*_*r*_*,* n = 12) were calculated using FSTAT Version 2.9.3^59^. Significant deviations from Hardy–Weinberg equilibrium, as indicated by deviations of *F*_*is*_ from zero, were tested by randomization using FSTAT. The frequencies of null alleles were estimated using CERVUS version 3.0 software^60^. Pairwise genetic distances based on a Jost’s *D* matrix among the 32 populations were calculated with GenoDive version 3.0 software^61^. To detect signatures of bottlenecks in the populations, we used the program INEST 2.2^62^ to assess excess heterozygosity in each population. We ran the model using 100,000 coalescent simulations, and tested significance using the Wilcoxon signed-rank test, calculated based on 1,000,000 permutations under both the stepwise mutation model (SMM) and the two-phase model (TPM).

### Analysis of population structure

Bayesian statistical inference of the population structure was performed by use of STRUCTURE 2.3.4 software^63^ with the correlated model for allele frequency using no prior population information (USEPOPINFO = 0), both with and without the LOCPRIOR model. The LOCPRIOR model allows for sample group information to be used to aid in the clustering process^64^. This model has been found to detect genetic structure at lower levels of divergence than the regular mode. The analysis was run 10 times for each value of *K* (number of inferred ancestral populations) from 2 to 8 for 1,000,000 iterations after a burn-in period of 1,000,000 iterations. Evanno et al*.*’s criterion of *∆K* was used to estimate the appropriate *K* value^65^. Furthermore, genetic variance among clusters was also used to perform AMOVA^66,67^ in GenoDive version 3.0 software^61^. Significance levels were determined using 10,000 permutations. Because the populations from Kyushu were clearly genetically distant from the others, the values of *∆K* and variance among clusters were also calculated using the 29 populations from outside of Kyushu. The CLUMPAK online tool^68^ was applied to calculate the *∆K* values and to graphically display the results produced by STRUCTURE. A neighbor-joining tree based on net nucleotide distances was generated using POPULATIONS v. 1.2.30^69^. The 618 accessions were used for the main analysis. For validation purposes, the 95 wild chestnut individuals collected by FTBC were co-analyzed with the 618 accessions. The results of the validation are shown in Supplementary Figure S2.

### Data analyses

To identify possible parent cultivars of wild individuals, putative parent–offspring relationships were calculated with CERVUS^60^ software, which uses a maximum-likelihood-based approach to infer parentage. The genotype data of the 30 nSSRs for 92 major cultivars (Supplementary Table S1) were used as the cultivar database of candidate parents. The paternity analyses were carried out to infer whether each wild accession had a parent–offspring relationship with each of cultivars. The parameters of the simulated genotypes were the following: “offspring” 100,000; “candidate parents” 100; “prop. sampled” 0.10; “prop. loci typed” 1.00; and “prop. loci mistyped” 0.02. Because the true parents of wild individuals could be outside of our database, we applied strict assignments using a 99% confidence threshold (95% for default parameter) for delta LOD scores to minimize the type-II error^69^ (when a paternity or maternity is attributed to a sampled individual, in this case a cultivar in the database, whereas the true parent is outside the set of sampled individuals). The average probability of non‐exclusion of an incorrect father using this approach was 1.5 × 10^−7^.

Assignment tests have been used to infer the origin of cultivars in several crops^70–72^. Assignment based on Bayesian criteria for computation for the 92 cultivars was carried out in GeneClass 2.0^73^ by using the wild populations as reference sets. We applied an exclusion test using the Monte Carlo resampling method^74^. The genotypes were generated by Markov Chain Monte Carlo simulations of 10,000 individuals for each of the wild populations. When the probability that the wild populations in a region were sources of a cultivar was less than a given threshold (α = 0.01), the populations in that region were excluded from candidate sources for the tested cultivar.

For the 26 wild populations on Honshu island, we tested isolation by distance. Mantel tests^75^ were used to estimate the correlation between (*F*_*ST*_/(1 − *F*_*ST*_)) and the geographic distances (km) between sampled populations based on 10,000 randomizations using GenAlEx v. 6.5 software. Because wild populations TSU and TOK carried substantial portions of the “cultivar” genetic cluster, correlation of genetic distance and *F*_*ST*_/(1 − *F*_*ST*_) was also calculated for pairs including either TSU or TOK.

## Supplementary Information

Supplementary Figures.

Supplementary Tables.
