# Supplementary material for: Genetic structure analysis of cultivated and wild chestnut populations reveals gene flow from cultivars to natural stands
Source: Sci Rep. 2021 Jan 8;11:240. doi: 10.1038/s41598-020-80696-1 (PMC7794426; doi:10.1038/s41598-020-80696-1)
Supplement: Supplementary file 1 — Supplementary Figures. [file 41598_2020_80696_MOESM1_ESM.pptx]

## Slide 1
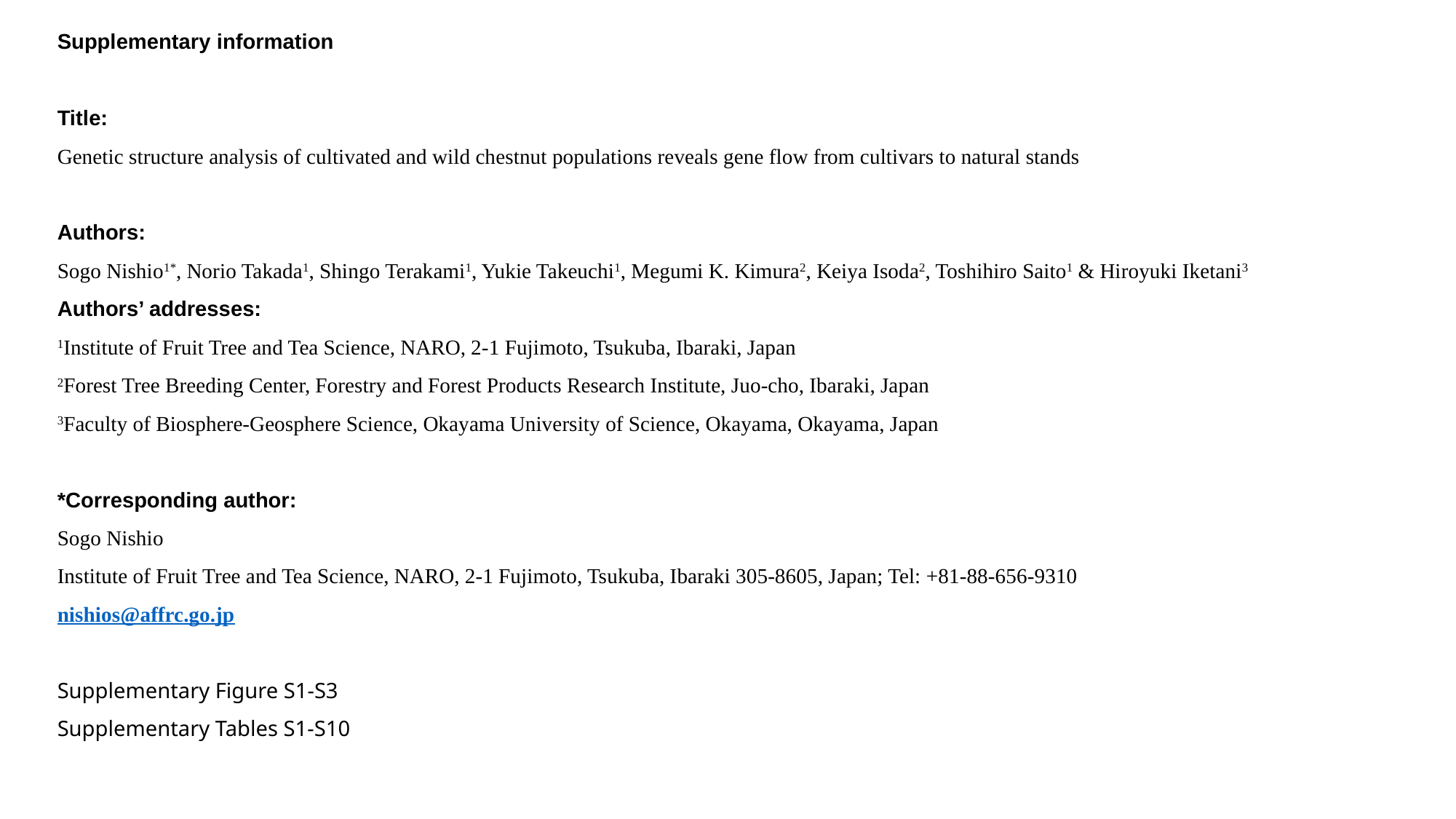

Supplementary information
Title:
Genetic structure analysis of cultivated and wild chestnut populations reveals gene flow from cultivars to natural stands
Authors:
Sogo Nishio1*, Norio Takada1, Shingo Terakami1, Yukie Takeuchi1, Megumi K. Kimura2, Keiya Isoda2, Toshihiro Saito1 & Hiroyuki Iketani3
Authors’ addresses:
1Institute of Fruit Tree and Tea Science, NARO, 2-1 Fujimoto, Tsukuba, Ibaraki, Japan
2Forest Tree Breeding Center, Forestry and Forest Products Research Institute, Juo-cho, Ibaraki, Japan
3Faculty of Biosphere-Geosphere Science, Okayama University of Science, Okayama, Okayama, Japan
*Corresponding author:
Sogo Nishio
Institute of Fruit Tree and Tea Science, NARO, 2-1 Fujimoto, Tsukuba, Ibaraki 305-8605, Japan; Tel: +81-88-656-9310
nishios@affrc.go.jp
Supplementary Figure S1-S3
Supplementary Tables S1-S10

## Slide 2
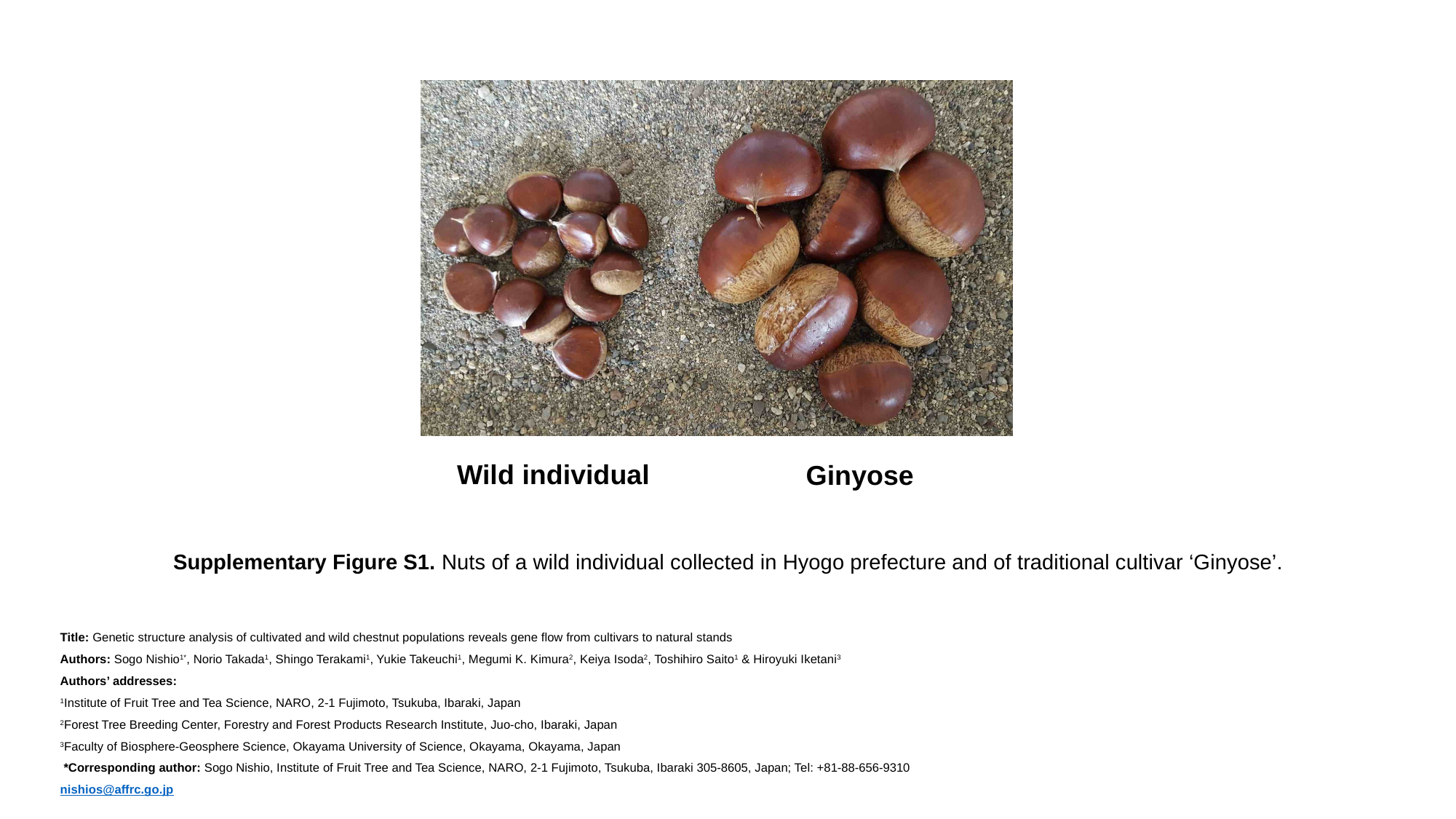

Wild individual
Ginyose
Supplementary Figure S1. Nuts of a wild individual collected in Hyogo prefecture and of traditional cultivar ‘Ginyose’.
Title: Genetic structure analysis of cultivated and wild chestnut populations reveals gene flow from cultivars to natural stands
Authors: Sogo Nishio1*, Norio Takada1, Shingo Terakami1, Yukie Takeuchi1, Megumi K. Kimura2, Keiya Isoda2, Toshihiro Saito1 & Hiroyuki Iketani3
Authors’ addresses:
1Institute of Fruit Tree and Tea Science, NARO, 2-1 Fujimoto, Tsukuba, Ibaraki, Japan
2Forest Tree Breeding Center, Forestry and Forest Products Research Institute, Juo-cho, Ibaraki, Japan
3Faculty of Biosphere-Geosphere Science, Okayama University of Science, Okayama, Okayama, Japan
 *Corresponding author: Sogo Nishio, Institute of Fruit Tree and Tea Science, NARO, 2-1 Fujimoto, Tsukuba, Ibaraki 305-8605, Japan; Tel: +81-88-656-9310
nishios@affrc.go.jp

## Slide 3
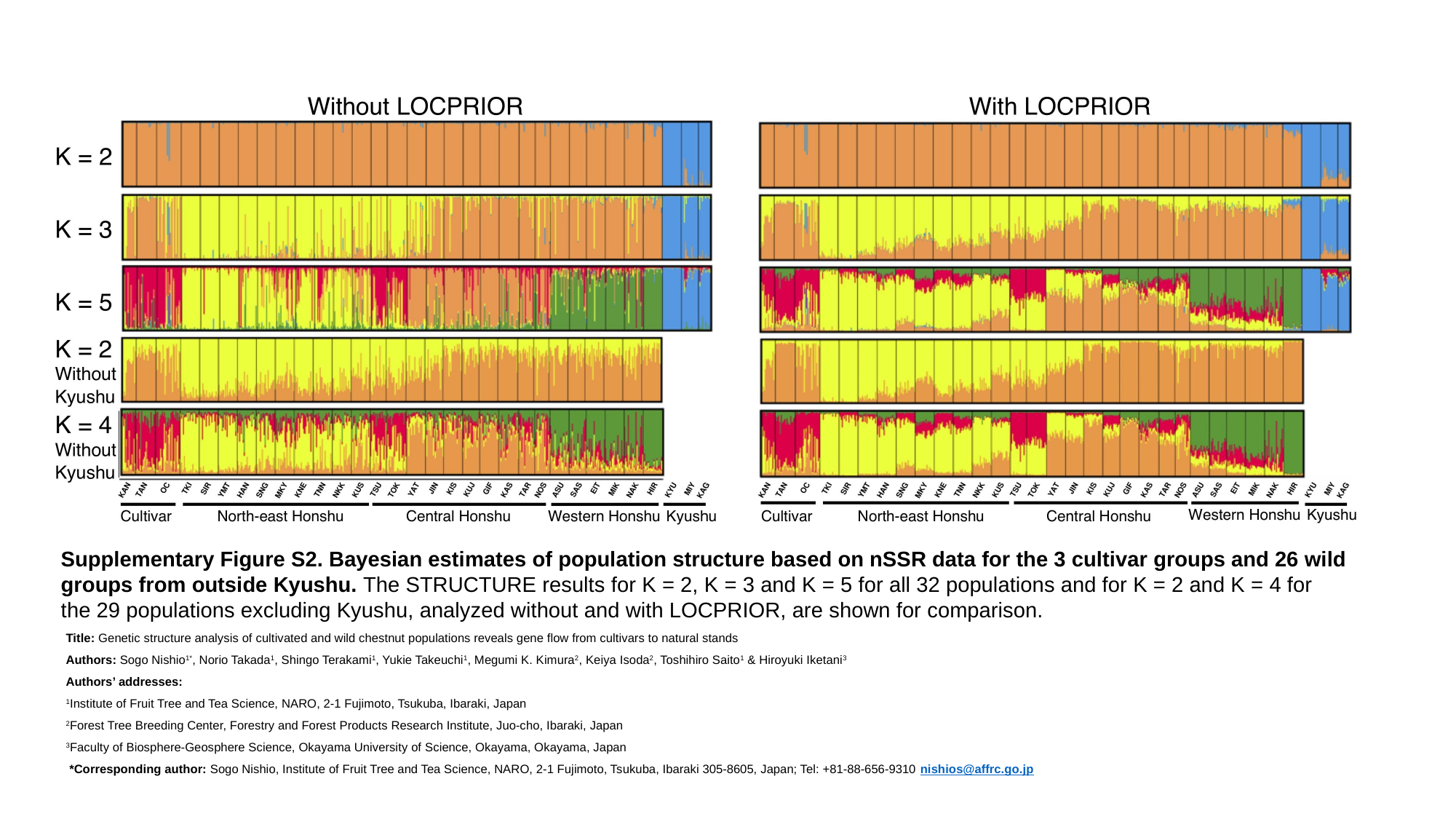

Supplementary Figure S2. Bayesian estimates of population structure based on nSSR data for the 3 cultivar groups and 26 wild groups from outside Kyushu. The STRUCTURE results for K = 2, K = 3 and K = 5 for all 32 populations and for K = 2 and K = 4 for the 29 populations excluding Kyushu, analyzed without and with LOCPRIOR, are shown for comparison.
Title: Genetic structure analysis of cultivated and wild chestnut populations reveals gene flow from cultivars to natural stands
Authors: Sogo Nishio1*, Norio Takada1, Shingo Terakami1, Yukie Takeuchi1, Megumi K. Kimura2, Keiya Isoda2, Toshihiro Saito1 & Hiroyuki Iketani3
Authors’ addresses:
1Institute of Fruit Tree and Tea Science, NARO, 2-1 Fujimoto, Tsukuba, Ibaraki, Japan
2Forest Tree Breeding Center, Forestry and Forest Products Research Institute, Juo-cho, Ibaraki, Japan
3Faculty of Biosphere-Geosphere Science, Okayama University of Science, Okayama, Okayama, Japan
 *Corresponding author: Sogo Nishio, Institute of Fruit Tree and Tea Science, NARO, 2-1 Fujimoto, Tsukuba, Ibaraki 305-8605, Japan; Tel: +81-88-656-9310 nishios@affrc.go.jp

## Slide 4
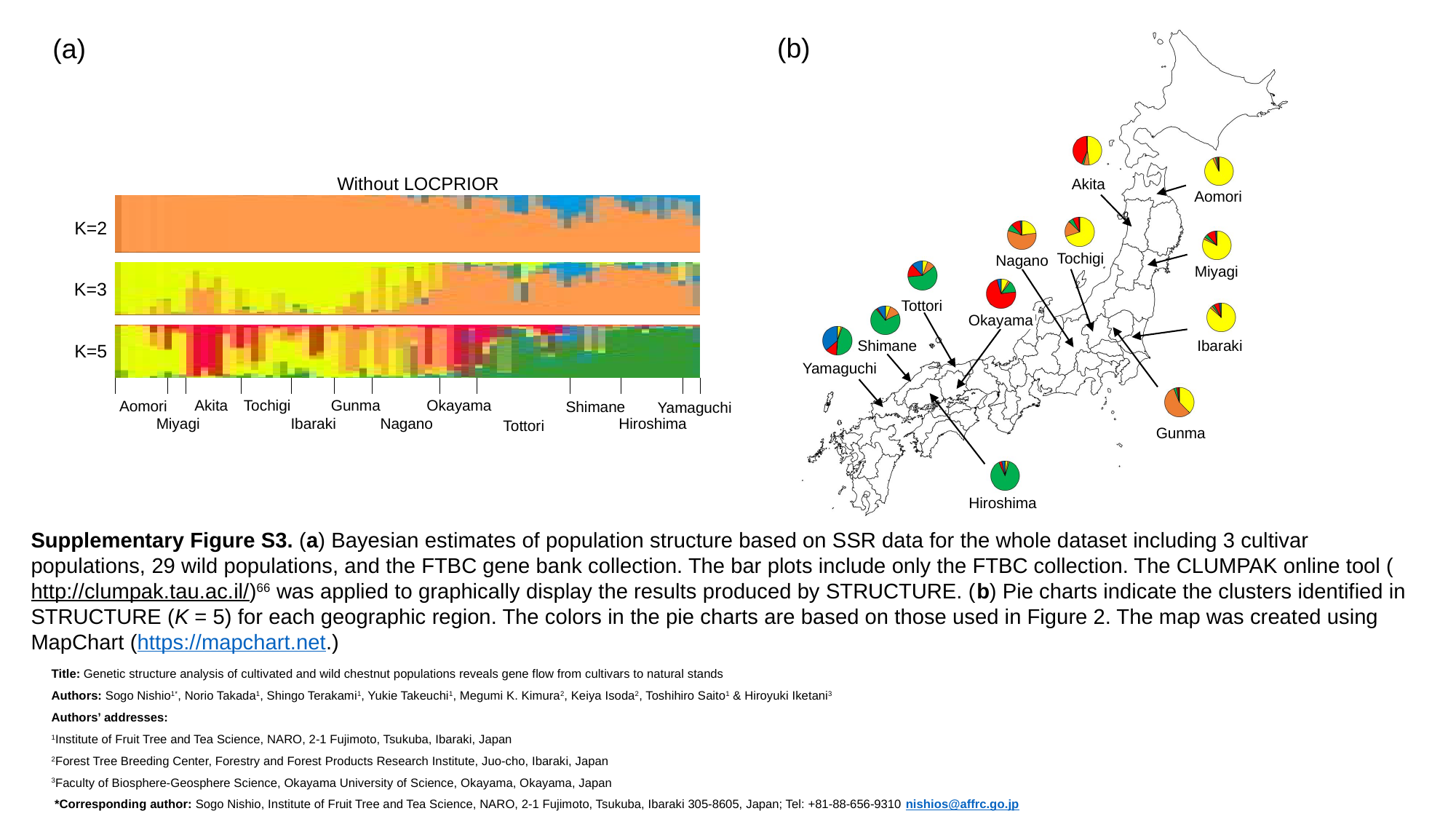

Aomori
Tochigi
Nagano
Miyagi
Tottori
Okayama
Shimane
Ibaraki
Yamaguchi
Gunma
(b)
(a)
Without LOCPRIOR
Akita
K=2
K=3
K=5
Akita
Tochigi
Okayama
Gunma
Aomori
Shimane
Yamaguchi
Miyagi
Ibaraki
Nagano
Hiroshima
Tottori
Hiroshima
Supplementary Figure S3. (a) Bayesian estimates of population structure based on SSR data for the whole dataset including 3 cultivar populations, 29 wild populations, and the FTBC gene bank collection. The bar plots include only the FTBC collection. The CLUMPAK online tool (http://clumpak.tau.ac.il/)66 was applied to graphically display the results produced by STRUCTURE. (b) Pie charts indicate the clusters identified in STRUCTURE (K = 5) for each geographic region. The colors in the pie charts are based on those used in Figure 2. The map was created using MapChart (https://mapchart.net.)
Title: Genetic structure analysis of cultivated and wild chestnut populations reveals gene flow from cultivars to natural stands
Authors: Sogo Nishio1*, Norio Takada1, Shingo Terakami1, Yukie Takeuchi1, Megumi K. Kimura2, Keiya Isoda2, Toshihiro Saito1 & Hiroyuki Iketani3
Authors’ addresses:
1Institute of Fruit Tree and Tea Science, NARO, 2-1 Fujimoto, Tsukuba, Ibaraki, Japan
2Forest Tree Breeding Center, Forestry and Forest Products Research Institute, Juo-cho, Ibaraki, Japan
3Faculty of Biosphere-Geosphere Science, Okayama University of Science, Okayama, Okayama, Japan
 *Corresponding author: Sogo Nishio, Institute of Fruit Tree and Tea Science, NARO, 2-1 Fujimoto, Tsukuba, Ibaraki 305-8605, Japan; Tel: +81-88-656-9310 nishios@affrc.go.jp
